# Diabetes-related instrument to assess preventive behaviors among adolescents (DIAPBA): a tool development and psychometric research

**DOI:** 10.1186/s12887-024-04632-2

**Published:** 2024-03-15

**Authors:** Ameneh Pooresmaeil Dorosteh, Mohtasham Ghaffari, Sakineh Rakhshanderou, Yadollah Mehrabi

**Affiliations:** 1 Ph.D of Health Education & Health Promotion, School of Public Health and Safety, Shahid Beheshti University of Medical Sciences, Tehran, Iran; 2 Professor of Health Education & Health Promotion, School of Public Health and Safety, Shahid Beheshti University of Medical Sciences, Tehran, Iran; 3 Associate Professor of Health Education & Health Promotion, School of Public Health and Safety, Shahid Beheshti University of Medical Sciences, Tabnak Ave., Daneshjou Blvd, P.O. Box 19835-35511, Tehran, Iran; 4 Department of Epidemiology, School of Public Health and Safety, Shahid Beheshti University of Medical Sciences, Tehran, Iran

**Keywords:** Adolescents, Behavior, Development, Psychometrics, Tool, Type 2 diabetes Mellitus

## Abstract

**Background:**

Type 2 diabetes is a chronic but preventable disease that is on the rise among adolescents. Evaluating adolescents’ behavior and planning to prevent it require a valid and reliable instrument. This study aims at designing a psychometric instrument to measure adolescents’ behavior with respect to type-2 diabetes.

**Research Design and methods:**

In this methodological research, 770 students (adolescent boys and girls aged 13–15 years) participated through multistage sampling. The Inclusion criteria were: junior high school students, students’ willingness for participation and not suffering from type-1 or type-2 diabetes. The questionnaire was designed by examining the relevant literature and the existing questionnaires as well as considering the research team’s comments. The validity of the study was determined through face validity and content validity both quantitatively and qualitatively. The construct validity was determined through exploratory and confirmatory factor analysis. Reliability was measured via intraclass consistency coefficient (ICC) and internal consistency reliability was measured by Cronbach Alpha. SPSS 16 and Eq. 6.1 were used for data analysis.

**Results:**

At first, a list of 47 initial items was designed and compiled, and after by removing similar (10 questions) or inappropriate sentences (12 questions), a draft questionnaire with 25 questions was designed. No items were removed in the face validity phase. Based on exploratory factor analysis, the number of items in the questionnaire was reduced to 20 items and was categorized in five dimensions of stress management, healthy food/healthy diet, unhealthy food/unhealthy diet, high-risk behavior, and self-care. The results of confirmatory factor analysis confirmed the model. The internal consistency coefficient was confirmed measuring Cronbach Alpha at 0.70 with ICC = 0.80.

**Conclusion:**

The questionnaire designed has standard psychometric properties to assess adolescents’ behavior with respect to type-2 diabetes prevention. The reliability and the validity of the questionnaire as well as its general structure were confirmed.

## Introduction

Diabetes is a chronic and metabolic disease that leads to high levels of blood sugar with type-2 diabetes as the most common one [1]. According to the International Federation of Diabetes, 463 million adults (20–79 years old) were suffering from type-2 diabetes in 2019. This figure is expected to rise to 783 million by 2045 [2]. In the Eastern Mediterranean region, Kuwait and Yemen recorded the highest (22%) and the lowest (3.9%) rate of type-2 diabetes respectively among adults of 20–79 years old in 2019. As one of the countries in the region, Iran had an outbreak record of 9.4% [3]. Type-2 diabetes was conceived to be rare among children and adolescents up until 30 years ago. However, in mid 1990s researchers saw a global increase in the number of cases among adolescents [1]. In addition, type-2 diabetes has been diagnosed on average among adolescents of 12–14 years of age [4]. In 2011, 1% of Iranian adolescents between 10 and 19 suffered from type-2 diabetes [5]. Unfortunately, new statistics are not available.

Type 2 diabetes can lead to many complications such as kidney complications, blindness, limb amputation, cardiovascular diseases, and stroke in the body. This disease can be more complicated and worrisome among children and adolescents than adults and has proven to be an aggressive disease with early side effects [6]. Adolescents with type 2 DM have very poor treatment outcomes and a rapid decline in their glycemic control with the current treatment protocols [7]. In addition, Patients with type 2 DM have a cluster of metabolic risk factors including obesity, Insulin Resistance (IR), hyperglycemia, dyslipidemia, hypertension, ectopic fat deposition and inflammation which predispose them to future cardiovascular disease (CVD) [8], This could potentially affect the economy of the nation apart from posing a large burden to the individual and his or her family [9].

Short-term complications of type 2 diabetes in children include diabetic ketoacidosis (DKA) and hyperosmolar hyperglycemic state (HHS) and Children may also present with acute decompensation in diabetic ketoacidosis (DKA) and/or hyperosmolar hyperglycemic state (HHS) [10]. A combination of factors such as socio-cultural, genetic and cultural factors causes type 2 diabetes [11]. The contributing factors to type-2 diabetes can be found in obesity, family history, ethnicity, sedentary lifestyle, history of diabetes during pregnancy, high-calorie diet, polycystic ovary syndrome (PCOS) [12], cholesterol and hypertension [13], addiction to TV (bingeing TV) [14], vitamin deficiency [15], smoking [16], alcohol [17], sleep deprivation [18], and stress and depression [19].

Health care systems worldwide such as on the affected individuals. To reduce this tremendous increase successful prevention methods are necessary [20]. Changing health behavior is an important step in disease prevention and management. Lifestyle-associated diseases are increasing because they are related to individuals’ behavior and the difficulty to influence them [21]. Behaviors are, in fact, observable actions or manners taken by people to respond to certain stimuli [22]. To curb the spread of diabetes, primary prevention should target adolescents [23]. Adolescence is a period beyond childhood when adolescents enter a new stage of life [24] and in this period, adolescents upgrade their skills and gradually assume more responsibility for their health [25]. Today, adolescents lead an unhealthy lifestyle, and unhealthy behavior can trigger type-2 diabetes [23].

Investigating diabetes-inducing behaviors and designing proper interventions are two important approaches to prevent and control type-2 diabetes. There is no precise instrument to assess preventive measures against this disease among adolescents. Studies that have attempted to design instruments mainly focus on diabetics’ quality of life (DQOL) [26], diabetic self-care [27], diabetic knowledge [28], and diabetics’ awareness, attitude and behavior [29, 30]. This study aimed at designing a reliable and valid instrument to assess adolescents’ preventive measures against type-2 diabetes to be used in education and prevention programs in the future.

## Methods

### Design and setting

This research is a methodological study conducted in Tehran in 2021. It followed a multistage sampling carried out on 770 adolescents between 13 and 15 (junior high school students).

### Inclusion and exclusion criteria

The participation criteria for the high school students were informed consent, voluntary participation and not suffering from type-1 or type-2 diabetes. Reluctance to participate in the study at any stage led to participants’ exclusion.

### Participants

The statistical population of this study was 770 adolescents aged 13 to 15 (boys and girls) from Tehran, who were selected through a multi-stage sampling. At first, Tehran was divided into five parts: North-East, South-East, Center, North-West and South-West, and one region was selected from each part. Then, a girl’s school and a boy’ school − 10 schools in total - were randomly selected from each region. Finally, 26 eligible students were selected randomly from each grade and completed questionnaire.

### Designing the instrument in four stages

#### Stage one

at this stage, the relevant questionnaires (32 cases), theses (12 cases), papers (31 cases) and the review of literature were used to design the questionnaire such as Menino et al. [31], Gillani et al. [32], and Fatema et al. [33].

#### Stage two

reviewing multiple instruments led to the extraction of relevant questions. A number of questions were designed through the existing papers and documents. Then, the experts added some more items to the pool of questions the first draft. Finally, the 47 initial items were designed and compiled, and after by removing similar (10 questions) or inappropriate sentences (12 questions), a draft questionnaire with 25 questions was designed.

#### Stage three

content validity, face validity, and construct validity were utilized to determine the validity of the instrument.

#### Content validity

Both quantitative and qualitative methods were employed to determine content validity. In quantitative analysis of content validity, content validity ratio (CVR) as well as content validity index (CVI) were calculated [34]. To determine content validity ratio, 11 experts (7 specialists in health education & health promotion, 2 endocrinologists and 2 pediatric endocrinologists) were asked to evaluate each question and comment on their significance as essential, effective, or non-essential. The following formula was used to determine the content validity ratio.

$$\text{C}\text{V}\text{R}=\frac{{\text{n}}_{\text{e}}-\raisebox{1ex}{$\text{N}$}\!\left/ \!\raisebox{-1ex}{$2$}\right.}{\raisebox{1ex}{$N$}\!\left/ \!\raisebox{-1ex}{$2$}\right.}$$

ne = The number of experts identifying an item as “essential”.

N = Total number of experts on the panel.

Finally, the resulting CVR amounts higher than 0.59 were accepted based on Lawshe Table. In addition, modifications were made to the questionnaire after negotiations and discussion with the research team. To determine the CVR for each item, the experts used the three criteria of ‘relevance’, ‘clarity’, and ‘simplicity’ to analyze each item through a 4-point Likert scale. The CVI result was determined by calculating the sum of scores for each item – 3 and 4 (the highest score) – as well as the following formula. The resulting CVI amounts higher than 0.79 were accepted. In the qualitative analysis, the experts were asked to evaluate each item.

$$\text{C}\text{V}\text{I}=\sum \frac{\text{N}\text{u}\text{m}\text{b}\text{e}\text{r}\ \text{o}\text{f}\ \text{a}\text{n}\text{s}\text{w}\text{e}\text{r}\text{s}\ 3\ \text{o}\text{r}\ 4 }{ \text{T}\text{o}\text{t}\text{a}\text{l}\ \text{N}\text{u}\text{m}\text{b}\text{e}\text{r}\ \text{o}\text{f}\ \text{a}\text{n}\text{s}\text{w}\text{e}\text{r} }$$

#### Face Validity

Face validity was determined both qualitatively and quantitatively.

#### Qualitative Face Validity

During this stage, to determine the qualitative face validity of the questionnaire, 20 students of 13–15 years of age (10 male and 10 female), who had been selected through multistage sampling, were interviewed face to face. Their views on the questions were sought with regard to levels of difficulty, consistency, and ambiguity. Finally, the necessary modifications were made considering the target group feedback and the research team.

#### Quantitative Face Validity

the quantitative face validity of the questionnaire was checked to remove the inappropriate questions and determine the significance of each question. The same 20 students were asked to examine the questions based on a 5-point Likert scale and select one.

1. Very important (5 points).
2. Important (4 points).
3. Rather important (3 points).
4. A little important (2 points).
5. Not important (1 point).

Then, the impact score of each question was calculated following this formula:

Impact score = Frequency (%) ×Importance the impact scores of higher than 1.5% were considered acceptable [35].

### Construct validity

The construct validity of questionnaire was performed with exploratory and confirmatory factor analysis. Kaiser-Mayer-Olkin (KMO) and Bartlett’s test of sphericity (BT) was used for the adequacy of samples to perform exploratory factor analysis.

For assess the fitness of the model in confirmatory factor analysis were used indices such as Adjusted Goodness of Fit Index (AGFI)، Root Mean Square Error of Approximation (RMSEA), Comparative Fit Index (CFI), Goodness of Fit Index (GFI). These indices determine the model fit and indicate how compatible a theoretical model is with an experimental model [36].

#### Stage four

to determine the reliability of the instrument, test-retest and internal consistency were employed.

Cronbach’s alpha was calculated to determine the internal consistency of the questionnaire items. To have good and sufficient internal consistency, Cronbach’s alpha should be 0.7–0.8 [37].

**Fig. 1:**
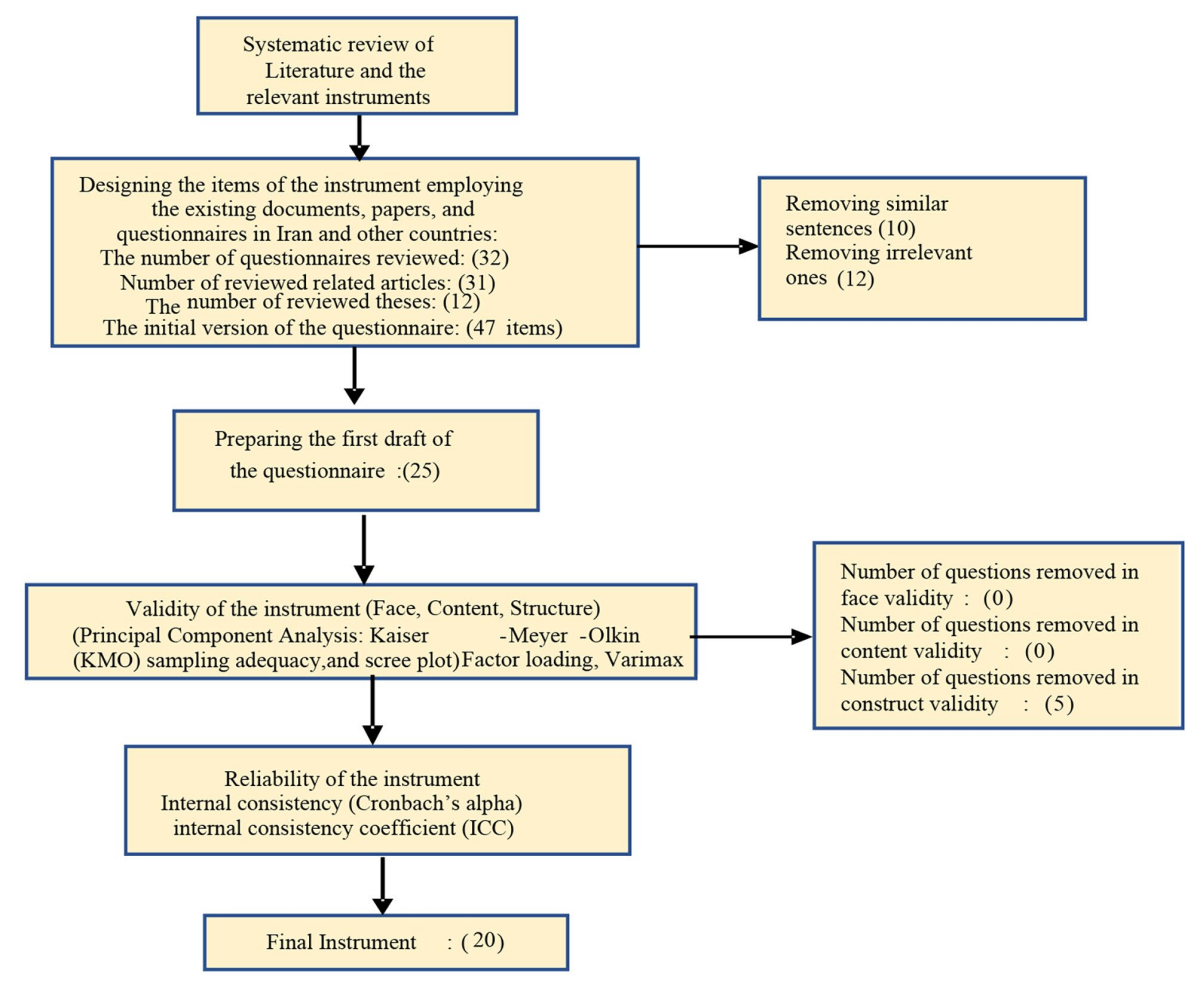
Flowchart of the design and psychometric stages of the questionnaire

Test-retest was employed to investigate the stability of the instrument over time. The questionnaire was completed by 40 adolescents (20 females and 20 males) within an interval of 15 days. A correlation coefficient of higher than 0.7 was considered acceptable for ICC (Internal Consistency Coefficient)) Fig. 1).

### Data analysis

The number and frequency of categorical variables, mean, and standard deviation of continuous and discrete variables were determined by descriptive statistics.

Content and face validity were determined with CVR /CVI and impact scores calculation respectively.

Construct validity was calculated through exploratory and confirmatory factor analysis. Cronbach’s alpha coefficient and the intraclass correlation coefficient (ICC) were calculated to determine the internal consistency and stability of the instrument respectively. Data analysis was conducted using SPSS16 and Eq. 6.1.

## Results

### The participants

There were 770 participants in this study. Some facts and figures about this population are as follows:

The mean and standard deviation of age was 13.99 ± 0.82. 59% of adolescents were female and 39% belonged to upper social economic class (Table 1).

**Table 1:** Demographic information of the Participants

Variables	Sub Group	Number	Percent
Age	13-year-old	267	35
	14-year-old	247	32
	15-year-old	256	33
Gender	Girl	453	59
	Boy	317	41
Grade of education	Seventh	270	36
	Eighth	242	31
	Ninth	258	33
Fathers’ occupation	Employee	223	29
	Self-employed	407	54
	Unemployed	48	6
	Retired	92	11
Mothers’ occupation	Employed	247	32
	House keeping	523	68
Fathers’ education	Illiterate	25	3
	Primary	58	7
	Intermediate	155	20
	Secondary	296	39
	Institutes/College	236	31
Mothers’ education	Illiterate	17	2
	Primary	72	9
	Intermediate	116	15
	Secondary	324	43
	Institutes/College	241	31
Economic situation	Poor	67	8
	Middle	268	35
	Good	304	39
	Excellent	131	17

### Designing the questions

Having explored various instruments and studied relevant papers and documents – questionnaires [34], papers [38], and theses [39] concerning type-2 diabetes – the research team came up with an initial list of 25 items.

### Content validity

At this stage, no item was removed. The average CVR and CVI were 0.80 and 0.87 respectively for the whole questionnaire.

### Qualitative face validity

In the qualitative phase of face validity, based on participants’ feedback some questions were modified, but no item was removed.

### Quantitative face validity

In the quantitative phase, the students’ responses were analyzed. Since the impact scores of all the questions were higher than 1.5, no question was removed at this stage. The questionnaire was ready to be checked for construct validity with 25 items.

### Construct validity

The appropriate sample size to use when conducting a factor analysis suggested minimums for sample size include from 3 to 20 times. In this study 20 samples were considered for each item and whit design effect 1.9 and %1 missing, 770 sample was calculated. 770 boy and girl students were selected by multistage sampling for construct validity (385 samples for EFA and 385 samples for CFA).

The KMO index and Bartlett’s test of sphericity showed the adequacy of the data for performing factor analysis. The KMO test result demonstrated the adequacy of the data (KMO = 0/78), so did the Bartlett’s test (p</001)). The results of exploratory factor analysis included the initial particular value, the particular value of extracted factors without rotation, and the particular value of extracted factors with rotation. Five factors with noticeable value (Eigenvalues) of above 1 were extracted. The first and fifth factors had particular values of 3.15 and 1.31 respectively. The first factor explains 15.75% of the variable’s variance and the sum of five factors explains 53.07% of the variable’s variance. Five items that loaded less than these amounts were removed from the questionnaire. Therefore, the questionnaire was reduced to 20 items.

The results of factor analysis with varimax rotation led to the extraction of five factors. The first factor (stress management) included six questions with the particular value of 3.55. Its factor loading was from the minimum 0.47 to maximum 0.80. The second factor (healthy food/healthy diet) included five questions with the particular value of 2.51. Its factor loading fluctuated between 0.56 and 0.68. The third factor (unhealthy food/unhealthy diet) included three questions with the particular value of 1.92, and the factor loading of 0.73 and 0.74. The fourth factor (high-risk behavior) included three questions with the particular value of 1.54, and the factor loading from 0.45 to 0.79. The fifth factor (self-care) included three questions with the particular value of 1.06, and the factor loading from 0.39 to 0.67 (Table 2). Five items that loaded less than the value of 0.4 were removed from the questionnaire. Therefore, the questionnaire was reduced to 20 items.

**Table 2:** Factor load of Behavior questionnaire items based on factor analysis with varimax rotatio

Rotated Component Matrix^a^					
	Component				
	F1	F2	F3	F4	F5
Planning and scheduling	0.80				
Practicing meditation and praying	0.76				
Positive thinking	0.71				
Regular exercise	0.70				
Participation in home and school activities	0.67				
Being in touch with friends, classmates, relatives and family members	0.47				
How much fruit do you eat every day? (One apple or one orange is equal to one unit)		0.68			
How much vegetable do you eat every day? (One glass of raw vegetable is equal to one unit of vegetable)		0.65			
How much milk and other dairy products (e.g., milk, yoghurt, ice cream) do you have every day? (One unit of dairy products is as much as one glass of milk or low-fat yoghurt, two matchboxes of cheese, or two glasses of lassi)		0.64			
How much meat and legume do you eat in a day? (One unit of meat is as much as 4 pieces of meat in food, half of a thigh or half of a breast, two boiled eggs, half of a glass of boiled legume, or a third of a cup of nuts)		0.60			
How much bread do you eat in a day? (One unit is as much as 30 g of bread or the size of a palm of hand, half a glass of rice or cooked spaghetti)		0.56			
How much fatty food (butter, bagel, cream, hamburger, sausage, and fried food) do you eat in a week?			0.74		
How much junk food (chips, corn, fruit juice, and soda) do you have in a week?			0.73		
How often do you eat fried food (fried chicken, fried fish, French fries, etc.)?			0.73		
How much time do you spend using TV, cell phone, tablet, or playing video games?				0.79	
Do you smoke?				0.77	
Do you smoke hookah?				-0.45	
How often do you take vitamin D supplement?					0.67
How often do you weigh yourself?					0.61
How much sleep do you get every night on average?					0.39
**Eigenvalues**	3.56	2.52	1.93	1.55	1.07
**% of Variance**	17.78	12.58	9.64	7.74	7.75
**Cumulative %**	17.78	30.36	39.99	47.73	53.07

**F1**: Stress Control **F2**: Healthy Nutrition **F3**: Unhealthy Nutrition **F4**: High-risk Behaviors **F5**: Self-Care

Analysis and description of behavior questionnaire items was performed. The mean, standard deviation, discriminatory power, difficulty index, Cronbach’s alpha if item deleted, communality and the range for each item was calculated. Discriminatory powers and difficulty indices were between 30 and 53 and 30–70 respectively that all were acceptable. (Table 3).

**Table 3:** Analysis and description of behavior questionnaire items

Items	Means	Standard deviations	Range	Discriminatory power	Difficulty indices	Cronbach’s alpha if item deleted	Communalities
Item1	2.57	1.21	4	0.49	0.55	0.66	0.65
Item2	2.46	1.32	4	0.53	0.54	0.65	0.62
Item3	2.18	1.32	4	0.49	0.43	0.66	0.55
Item4	2.79	1.15	4	0.43	0.63	0.66	0.54
Item5	2.18	1.29	4	0.46	0.42	0.66	0.48
Item6	2.51	1.18	4	0.37	0.52	0.67	0.43
Item7	1.80	0.85	3	0.39	0.63	0.69	0.55
Item8	2.09	1.07	4	0.32	0.58	0.70	0.51
Item9	1.79	0.90	3	0.39	0.61	0.68	0.47
Item10	1.64	1.22	4	0.32	0.30	0.70	0.52
Item11	1.64	1.54	4	0.30	0.38	0.69	0.41
Item12	2.13	1.08	4	0.31	0.40	0.69	0.58
Item13	2.49	1.20	4	0.37	0.59	0.68	0.63
Item14	2.43	1.16	4	0.41	0.54	0.69	0.55
Item15	3.43	1.04	4	0.39	0.70	0.69	0.72
Item16	3.44	1.03	4	0.41	0.70	0.69	0.68
Item17	2.08	1.00	4	0.31	0.33	0.70	0.42
Item18	2.34	1.40	4	0.33	0.52	0.70	0.51
Item19	2.25	0.87	2	0.43	0.53	0.68	0.44
Item20	2.51	1.32	4	0.41	0.54	0.68	0.36

Correlation coefficients among stress control, healthy nutrition, unhealthy nutrition, high risk behaviors and self-care were calculated using Pearson correlation test (Table 4).

**Table 4:** Correlations between the identified factors in EFA

	Stress Control	Healthy Nutrition	Unhealthy Nutrition	High risk Behaviors	Self-Care
Stress Control	1				
Healthy Nutrition	0.125^**^	1			
Unhealthy Nutrition	0.077^*^	− 0.013	1		
High risk Behaviors	0.153^**^	− 0.191^**^	0.334^**^	1	
Self-Care	0.384^**^	0.055	− 0.023	0.120^**^	1

**. Correlation is significant at the 0.01 level (2-tailed)

*. Correlation is significant at the 0.05 level (2-tailed)

**Fig. 2:**
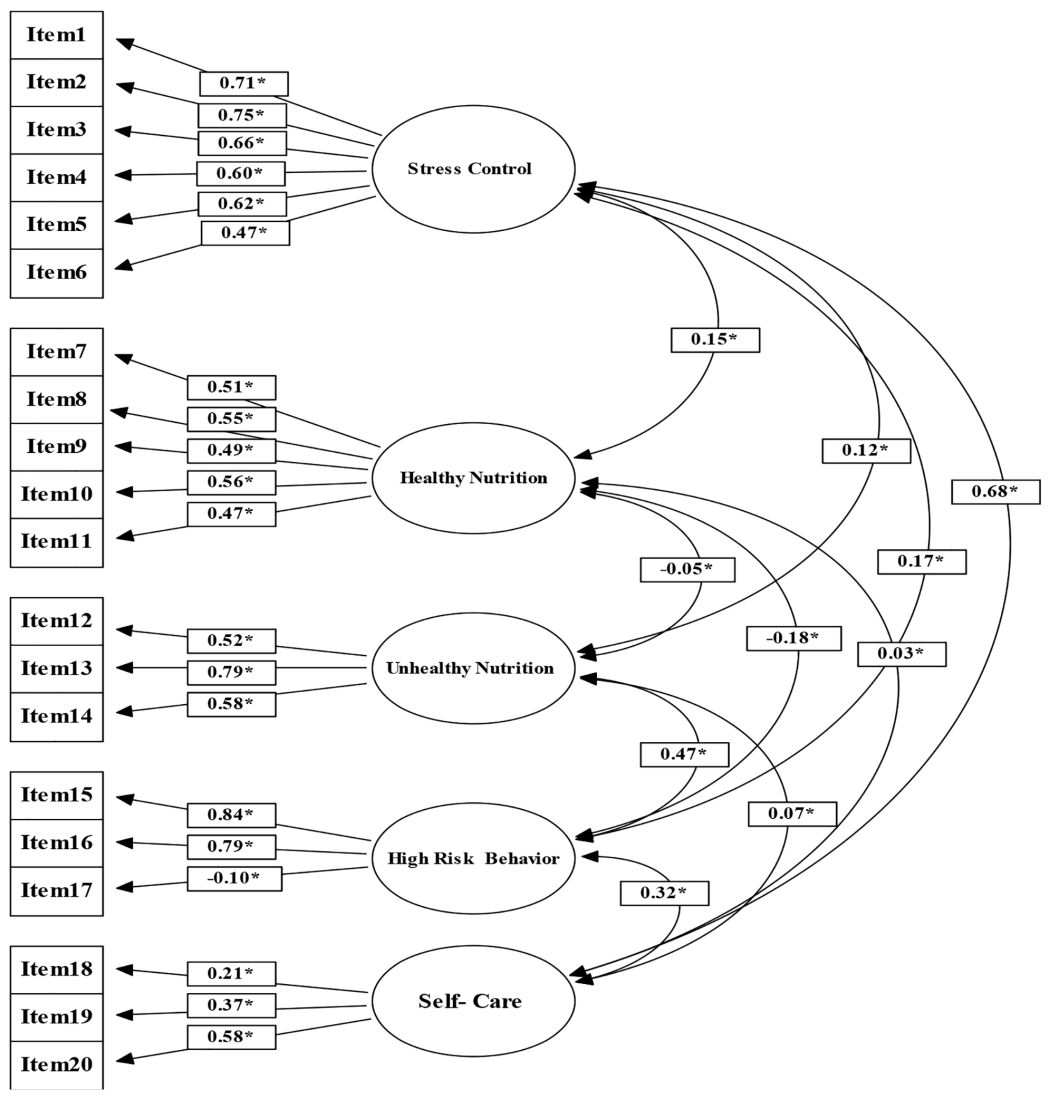
Diagram of confirmatory factor analysis

In the present study, the results of the confirmatory factor analysis as well as the following values were obtained (Fig. 2). Chi-Square to DF = 3.5, RMSEA = 0.05, RMR = 0.06, AGFI = 0.91, GFI = 0.93 and CFI = 0.92. And, according to Table 5, the results of confirmatory factor analysis were acceptable.

**Table 5:** Assess the fitness of the model

Model fitness indicators	Indexes values
Chi-Square	546.965
Degrees of Freedom (DF)	155
Root Mean Square Error of Approximation (RMSEA)	0.05
Root Mean-square Residual (RMR)	0.06
Adjusted Goodness of Fit Index (AGFI)	0.91
Goodness of Fit Index (GFI)	0.93
Comparative Fit Index (CFI)	0.92

### Reliability

The internal consistency of the instrument using Cronbach’s alpha coefficient turned out to be 0.70 for the whole instrument (between 0.70 and 0.80). The stability of the instrument using Intraclass Correlation Coefficient –ICC in a 15-day period was 0.80 for the whole instrument, fluctuating between 0.70 and 0.80 for the factors in the instrument (Table 6).

**Table 6:** The ICC and the Cronbach’s α coefficient of each factor and the whole of questionnaire

Factor	Number of Items	Cronbach’s alpha coefficient	Intra-class Correlation Coefficient ICC (*N* = 40)
Stress Control	6	0.80	0.80
Healthy Nutrition / Healthy Diet	5	0.70	0.77
Unhealthy Nutrition / Unhealthy Diet	3	0.70	0.78
High-risk Behaviors	3	0.70	0.70
Self-Care	3	0.70	0.70
Total	20	0.70	0.80

### The final instrument

The approved questionnaire included 20 items and had five dimensions (based on the type and content of the questions): stress management, healthy food/healthy diet, unhealthy food/unhealthy diet, high-risk behavior, and self-care. The range of scores given to questions regarding adolescents’ behavior was between 0 and 4 as well as between 1and 5. The highest attainable score was 82 and the lowest was 0. The areas of the questionnaire, the number of questions, options, and the range of score changes are shown in Table 7.

Finally, in order to measure the Behaviors of 770 adolescents, a questionnaire made with porslin (https://survey.porslin.ir/s/d1KMSO) was sent to the samples (due to the corona pandemic). Then the collected data was entered into spss16 and analyzed.

**Table 7:** The questionnaire areas, number of questions, options and range of score changes

Area	Number of questions	Options	Variation range
Stress Control	6 questions	Always/Most of the time/Sometimes/Rarely/Not at all	0–24
Healthy Nutrition / Healthy Diet	2 questions 2 questions 1 question	2 units/2–3 units/3–4 units/5 − 4 units/I do not consume at all 1 unit/2 units/3 units/4 units/I don’t consume at all 1–2 units/5 − 3 units/8 − 6 units/11 − 9 units/I don’t consume at all	0–8 0–8 0–4
Unhealthy Nutrition / Unhealthy Diet	3 questions	Every day of the week / 5–6 times a week / 4 − 3 times a week / 1–2 times a week / I don’t eat at all	0–12
High-risk Behaviors	1 question 2 questions	Less than 1 h/1–2 h/2–3 h/more than 3 h/I don’t use TV, tablet, mobile phone or electronic games at all Always/Most of the time/Sometimes/Rarely/Not at all	1–5 0–8
Self-Care	1 question 1 question 1 question	One vitamin D supplement per week / one every 2–3 weeks / one per month / one per year / I don’t take it at all Once a month/once every 2–3 months/once every 6 months/once a year/I don’t measure at all Less than 7 h/7–8 h/more than 8 h	0–4 0–4 1–3
Total	20 questions	Different options	(0–72),(1–5), (1–3)

## Discussion

This study aimed at designing a psychometric instrument for assessing adolescent behavior regarding type-2 diabetes. Content validity, face validity, and construct validity were calculated to meet the scientific requirements of the research study. As content validity is a prerequisite for other kinds of validity as well as a crucial step in the process of designing the questionnaire, one method of calculating content validity, that is CVR (content validity ratio) and CVI (content validity index) were used. In this study based on Lawshe Table, the CVR above 0.59 and CVI above 0.79 were accepted. Face validity was determined both qualitatively and quantitatively. Face validity is attained when the instrument is superficially in line with the purpose of the study. It also seeks to find out whether the participants of the study agree with the expressions and sentences used in the questionnaire [40]. In the quantitative phase, the impact score of all the items was above 1.5; therefore, all the items were kept as proper for the next round of analysis. The results indicate that the expressions used have been relevant and significant. In the qualitative analysis of face validity, some questions and items needed some minor modifications that were introduced by students’ views.

In this research, factor analysis was used to determine internal coherence as well as the construct validity of the questionnaire. Exploratory factor analysis and confirmatory factor analysis were used to determine construct validity. The KMO value for all the constructs was 0.78 and the significance level in the Bartlett’s test of sphericity (BT) was 0.001 indicating the adequacy of sampling for factor analysis. The construct validity of the instrument to assess adolescents’ behavior regarding type-2 diabetes was obtained by exploratory factor analysis. This analysis gave rise to the extraction of five factors: stress management, healthy food/healthy diet, unhealthy food/unhealthy diet, high-risk behavior, and self-care.

Stress is believed to be one of the major factors negatively affecting our health. High stress levels have shown to be strongly associated with many physical and emotional problems, such as cardiovascular disease, chronic pain, anxiety disorders, depression, burnout, and addictions [41]. Adolescence is a critical stage of life when adolescents face greater stress, extreme vulnerability to mental, social, and biological pressure with a decrease in disease resistance [42]. Over 400 years ago, a distinguished British physician, Thomas Willis, asserted that diabetes is especially prevalent among people who experience stress, melancholia, or long-term grieving. Numerous studies also point out that stress can contribute to type-2 diabetes [43]. Therefore, training adolescents how to deal with or cope with stress plays a significant role in preventing or delaying type-2 diabetes.

The other factors extracted in the present study are healthy food and unhealthy food. According to worldwide research on diet and eating in 188 countries, there is a close interaction between diet, disease and death. The significance of diet in managing and preventing type-2 diabetes is seen in its effect on weight and metabolic control [44]. The Western Pattern Diet (WPD) or Standard American Diet (SAD) during adolescence can increase the likelihood of type-2 diabetes. Due to the interaction between the nutrients and the physical properties of food, the general pattern of diet can increase the risk of type-2 diabetes more than the constituent parts [45]. Therefore, adolescents are prone to type-2 diabetes in the future if unhealthy eating habits persist. What schools and communities need to do is promote healthy eating habits among adolescents. These programs aim at training parents and adolescents to identify high risk factors regarding T2DM and to encourage preventive behavior and habits.

The fourth factor in this study is self-care. It is defined as any intentional attempt for physical, psychological, and emotional care. The dimensions of self-care include food, exercise, medicine, emotions, sleep, and medical care [46]. Improving the quality of sleep, changing sedentary lifestyle or getting more physical exercise [47] and generally adopting a healthy lifestyle as examples of self-care can significantly reduce the risk of type-2 diabetes among adolescents. Self-care as a means to prevent type-2 diabetes requires fundamental environmental changes, which calls for cooperation among all the stakeholders as parents, schools, teachers, healthcare providers, food industries, entertainment industry, economists, policymakers, and state organizations.

The simplest and easiest method of confirmatory factor analysis shows that the closer AGFI to 1, the fitter the model, as a result confirming the hypothesized relationships between factors.

In other words, AGFI above 0.90 demonstrates an acceptable value of Model-Fit.

In addition, RMSEA (root mean square error of approximation) should be preferably close to zero. RMSEA lower than 0.10 demonstrates an acceptable value of Model-Fit [48]. In this study, Eq. 6.1 showed AGFI to be 0.91, which also demonstrates an acceptable value of Model-Fit. RMSEA equaled 0.05 and the ratio of Chi-Square to df was 3.5.

Cronbach’s Alpha Coefficient was calculated to measure internal consistency reliability of the whole instrument as well as its different areas. The internal consistency of the final version of the instrument proved its reliability. The commonly used method for measuring internal consistency is by calculating the Cronbach Alpha coefficient and according to expert suggestions, the Cronbach’s alpha value is expected to be at least 0.70 to indicate adequate internal consistency of a given questionnaire [49]. In addition, test-retest method was used to determine the consistency of the instrument. Test-retest reliability involves administering the questionnaire to the same group of respondents at different point of time and repeating the research. The results are then compared for similarity to decide the reliability [49]. Based on the results, the instrument proved to be consistent.

### Strengths and limitations of the study

The distribution of the sample in the population in terms of number provides the generalizability of the data, making it one of the strengths of this research. In addition, the instrument designed has an acceptable level of validity and reliability that can be employed by researchers and experts in the related fields. One of the limitations of the study is the method of self-report used in completing the questionnaire. This method makes it difficult to compare the differences observed among individuals or different subgroups. Another limitation of this study was the lack of discriminant and convergent validity studies.

### Suggestions

Since the psychometric properties of the instrument were statistically verified, this instrument is recommended as a standard method to be used in studies in the area of preventive behavior against type-2 diabetes.

## Conclusion

In this study, an instrument was designed to measure adolescents’ behavior of type-2 diabetes in Tehran. Data analysis approved the content validity, face validity, construct validity, internal consistency, and stability of the instrument. This instrument is an objective, simple yet comprehensive tool to assess adolescents’ behavior of type-2 diabetes, which can be used in future research projects.

## Data Availability

All data relevant to the study are included in the article.
